# Cytomegalovirus Infection in Patient with Clear Cell Renal Cell Carcinoma

**DOI:** 10.1155/2023/5560673

**Published:** 2023-11-13

**Authors:** Ikhwan Rinaldi, Abdul Muthalib, Januar Widodo Sutandar, Hendro Adi Kuncoro, Bambang Irawan Harsono, Nelly Susanto, Tjondro Setiawan, Kevin Winston, Idham Rafly Dewantara, Ihya Fakhrurizal Amin, Yuli Maulidiya Shufiyani

**Affiliations:** ^1^Division of Hematology and Medical Oncology, Department of Internal Medicine, Cipto Mangunkusumo National General Hospital, Faculty of Medicine, Universitas Indonesia, Jakarta, Indonesia; ^2^Department of Internal Medicine, Gading Pluit Hospital, Jakarta, Indonesia; ^3^Department of Cardiology, Gading Pluit Hospital, Jakarta, Indonesia; ^4^Department of Pulmonology, Gading Pluit Hospital, Jakarta, Indonesia; ^5^Department of Radiology, Gading Pluit Hospital, Jakarta, Indonesia; ^6^Faculty of Medicine, Universitas Indonesia, Jakarta, Indonesia

## Abstract

**Introduction:**

Cytomegalovirus (CMV) infection is a widespread condition that can affect individuals of all ages. Most cases of CMV infection are mild and resolve on their own. However, in immunocompromised individuals, such as post-transplant patients or those with cancer, severe infections can occur. While there have been several studies on CMV infection in post-transplant patients, there is limited literature on CMV infection in cancer, particularly in kidney cancer. *Case Report*. In this case report, we present the case of a 61-year-old man with clear cell renal cell carcinoma who underwent targeted therapy with the receptor tyrosine kinase (RTK) inhibitor lenvatinib and the mammalian target of rapamycin (mTOR) inhibitor everolimus. The patient was hospitalized for 26 days and admitted to the intensive care unit (ICU) due to shortness of breath, decreased oxygen saturation, and irregular breathing. Cytomegalovirus polymerase chain reaction (PCR) test results were positive. Given the high prevalence of CMV infection in developing countries, it is likely that the patient had a reactivation of CMV. As such, the patient was subsequently treated with ganciclovir for 14 days and showed improvement in symptoms such as shortness of breath, cough, fever, and increased oxygen saturation. Following recovery, the patient received maintenance therapy with oral valganciclovir for 7 days. No further symptoms appeared during subsequent cancer treatments.

**Conclusion:**

Cancer patients who are undergoing treatment are at a higher risk for developing opportunistic infections, which can result in morbidity and mortality. Therefore, healthcare professionals should be aware of the possibility of CMV infection in cancer patients and be prepared to diagnose and treat the infection, particularly in areas where the prevalence of CMV infection is high.

## 1. Introduction

Cancer patients face a heightened risk of infections due to their compromised immune system. Among patients with solid tumors, the primary source of infection is often the resident microflora, with Gram-positive bacteria being the leading cause [1]. In addition, cytomegalovirus (CMV) infection emerges as a significant contributor to infections in cancer patients.

Cancer treatment is linked to a heightened risk of infection, including CMV. Notably, the use of phosphatidylinositol-3 kinase (PI3K) inhibitors is associated with an elevated risk of pneumocystis pneumonia (PCP) and reactivation of CMV [2]. A case report by Modvig et al. described a patient with non-Hodgkin's lymphoma developing severe CMV reactivation after chemotherapy [3]. Other viruses such as hepatitis B and varicella-zoster infection can reactivate after the use of everolimus [2]. Smaller increased risks of infection have also been observed in patients taking vascular endothelial growth factor receptor (VEGFR) agents [2]. Therefore, it is crucial that cancer patients undergoing treatment are regularly monitored for early signs of infections to prevent complications. Studies indicate that over 90% of cancer patients have positive CMV infection, making CMV reactivation a potential complication of cancer therapies [4, 5].

Currently, there is limited research on the prevalence of CMV infection in cancer patients, with most of the available literature being case reports and small studies. However, there are no reports specifically documenting CMV infection in patients with renal cancer. In this case report, we present the case of a patient with clear cell renal cell carcinoma who developed symptoms of shortness of breath as a result of CMV reactivation after receiving targeted cancer therapies.

## 2. Case Presentation

A 61-year-old male patient presented to the emergency room in June 2021, with the primary complaint of generalized weakness over the past week, accompanied by fever, nausea, vomiting, and diarrhea. Vital signs of the patient were as follows: blood pressure of 120/80 mmHg, heart rate of 102 beats per minute, respiratory rate of 26 breaths per minute, body temperature of 38.3°C, and oxygen saturation of 92% on room air. Physical examination and chest X-ray examination revealed no abnormalities.

Initial blood test results showed the following: hemoglobin 13.5 g/dL, hematocrit 39%, white blood cell count 10.1 × 10^3^/*µ*L, blood glucose 173 mg/dL, sodium level 132 mEq/L, potassium 3.6 mEq/L, and lactate dehydrogenase 720 U/L. Kidney and liver function tests were within the normal range. The patient was hospitalized and subsequently given metamizole every 8 hours and a single dose of intravenous moxifloxacin.

**Figure 1:**
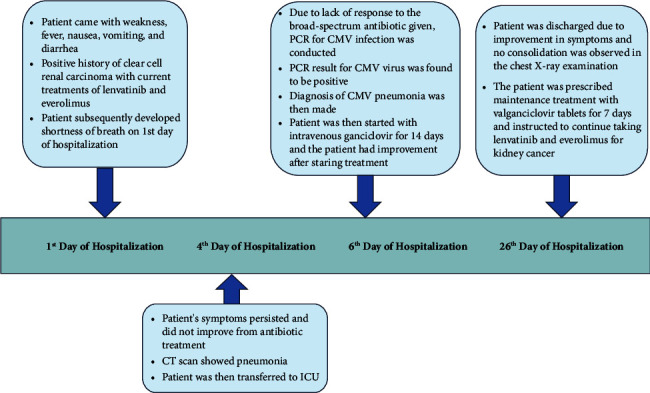
Case report timeline.

The patient's past medical history was significant for a clear cell renal cell carcinoma diagnosis made in June 2019. A brain magnetic resonance imaging (MRI) performed in April 2021 revealed a suspected dural metastatic mass in the right tentorium cerebelli. The patient had undergone several treatment modalities, including cryosurgery, a TAC (taxotere, adriamycin, and cyclophosphamide) regimen, and targeted therapies such as pembrolizumab, axitinib, pazopanib, tracetat, lenvatinib, and everolimus. At the time of presentation, the patient was receiving lenvatinib and everolimus as targeted therapy for their renal cancer. Timeline of the patient can be seen on Figure 1.

**Figure 2:**
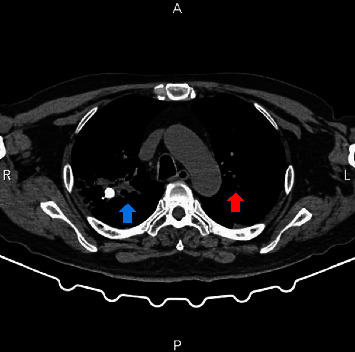
Thorax-computed tomography shows infiltrate in the upper right lobe (blue arrow) and the upper left lobe (red arrow).

On the first day of hospitalization, the patient developed a high fever up to 39°C, shortness of breath, and a decreased oxygen saturation of 88% on room air. Laboratory tests revealed a procalcitonin level of 0.73 ng/ml (normal <0.1) and a C-reactive protein (CRP) level of >200 mg/dL (normal <0.5). A PCR test for severe acute respiratory syndrome coronavirus 2 (SARS-CoV-2) was conducted to investigate the possibility of coronavirus disease 2019 (COVID-19) pneumonia infection, which turned out to be negative. A blood culture also returned negative results. Further investigation using multislice computed tomography (MSCT) revealed ground-glass opacity with crazy paving and infiltrates in the right upper lobe and lung base areas, as well as glass opacity in the left upper lobe lung area, as shown in Figure 2. The patient was initially suspected of having community-acquired pneumonia and was started on double strength antibiotics of trimethoprim/sulfamethoxazole.

During treatment, the patient's symptoms persisted and did not improve. The patient continued to experience fever, shortness of breath, low oxygen saturation, and irregular breathing patterns, leading to their admission to the ICU on the fourth day of treatment. The patient's vital signs were blood pressure of 110/70, heart rate of 126 beats per minute, respiratory rate of 30 breaths per minute, and body temperature of 38.4°C. Additional imaging, such as an anterior to posterior (AP) chest X-ray, revealed enlarged hilar, consolidation in both lung fields, multiple calcifications in the upper right lung fields, dullness in the costophrenic sinuses, and mild bilateral pleural effusions. Laboratory results on the 4^th^ day of hospitalization are presented in Table 1.

**Figure 3:**
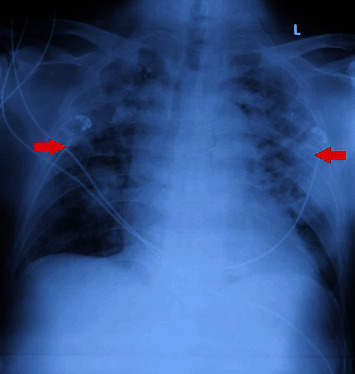
Chest X-ray shows both consolidations in both lung fields (red arrow).

The patient's symptoms persisted despite the administration of parental antibiotics consisting of meropenem, levofloxacin, and moxifloxacin. The patient continued to experience fever, shortness of breath, and decreased oxygen saturation, despite receiving nonrebreathing oxygen at a flow rate of 12 LPM. A subsequent chest X-ray revealed persistent consolidation in both lung fields, as depicted in Figure 3.

From the patient's symptoms of shortness of breath and the lack of response to the broad-spectrum antibiotic therapy given, we suspected an infection from *Pneumocystis jirovecii* pneumonia (PJP) and CMV infection. Thus, we performed both CMV and PJP PCR examinations using blood specimens on the fifth day of treatment. The PCR examination showed positive CMV PCR and negative PJP PCR results. Thus, the patient had pneumonia from CMV infection.

The patient subsequently received intravenous ganciclovir at a dose of 10 mg/kg/24 hours for 14 days. A blood test revealed a D-Dimer level of 3480 *μ*g/ml (reference range: 0–0.23). Sputum examination and throat swab culture were performed due to the patient's acute productive cough. The sputum culture revealed the presence of bacteria, leukocytes, epithelium, *Candida albicans*, and *Acinetobacter baumannii* colonies. The throat swab examination revealed the presence of Gram-positive coccus (1+). The treatments with intravenous meropenem and intravenous levofloxacin were then discontinued and replaced with intravenous piperacillin + tazobactam.

Despite treatments, the patient continued to experience fever up to 38°C, productive cough, and shortness of breath initially. The patient's symptoms of fever, cough, shortness of breath, and decreased oxygen saturation gradually improved as they neared the end of the 14-day ganciclovir treatment. The blood test revealed that CRP and procalcitonin levels decreased.

The patient was discharged from the hospital on the 26th day of hospitalization, after showing improvement in symptoms, and no consolidation was observed in the chest X-ray examination. The patient was prescribed maintenance treatment with valganciclovir tablets for 7 days and instructed to continue taking lenvatinib and everolimus for their kidney cancer. The patient was scheduled for regular follow-up visits and underwent chemotherapy until March 2022. During this period, the patient did not show any signs of recurrent CMV infection.

## 3. Discussion

CMV infection exhibits a substantial global prevalence. Epidemiological literature indicates that in developed nations, the prevalence stands at approximately 50%, whereas in developing countries, it can escalate to nearly 100% [6, 7]. There are two sources of CMV infection: direct acute infection, which occurs during the primary infection, and reactivation of a latent infection, which is commonly seen in immunosuppressed individuals [8]. The reactivation of CMV, as observed in cancer patients, can contribute to an elevated risk of mortality [9].

The precise mechanism of CMV reactivation remains incompletely understood. Nevertheless, researchers speculate that tumor necrosis factor (TNF) might play a role in this process. TNF-alpha is believed to bind to the TNF receptor on latently infected cells, leading to the activation of protein kinase C and nuclear factor kappa B (NF-*κ*B) [10–13]. Consequently, this activation triggers the transcription of the Human Cytomegalovirus Immediate-Early (HCMV) IE gene and initiates viral replication [10–13]. In addition, it is thought that epinephrine, catecholamines, and norepinephrine may increase the concentration of cyclic adenosine monophosphate (cAMP), which can stimulate the IE enhancers/promoters, leading to HCMV reactivation [10, 11]. As previously noted, CMV infection and reactivation are frequently linked to immunocompromised states, including acquired immune deficiency syndrome (AIDS), cancer, and extended steroid therapy. It is important to highlight that individuals undergoing initial treatment for cancer with cytotoxic anticancer agents could encounter additional suppression of their immune systems.

Most studies investigating the state of immunosuppression and CMV reactivation have primarily focused on organ transplant recipients and patients with AIDS [14, 15]. However, there are several studies that have analysed CMV reactivation in cancer patients although the number of published studies is still limited [16]. A study by Prabhash et al. showed that out of 73 patients with solid malignancies, at least 30 tested positive for CMV PCR, indicating that patients with solid tumors have an immunosuppressive state [17]. A prospective cohort study by Schlick et al. detected that out of 107 cancer patients with concomitant CMV infection, 17 had solid cancers [18]. Of these 17 patients, 15 underwent palliative chemotherapy and 5 underwent adjuvant chemotherapy. This highlights that not only blood cell malignancies but also solid tumors have a potential for CMV reactivation which carries a high risk.

The patient in this case is a 61-year-old male who was diagnosed with clear cell renal cell carcinoma in 2019. In a study conducted by Dziedzic et al., it was found that age is an important risk factor for CMV infection [19]. In addition, lymphopenia and T-cell depletion have also been identified as significant risk factors for CMV infection [19].

There are no distinctive symptoms exclusively associated with CMV infection, making its diagnosis potentially challenging. Primary CMV infection frequently manifests as either asymptomatic or with subclinical symptoms. The observed symptoms bear resemblance to those of other infections, notably including fever. In cases involving immunocompromised individuals, this fever might be prolonged [20]. The clinical manifestations of CMV infection in immunocompromised patients can depend on the organ that is infected.

The current gold standard for CMV diagnosis is a histopathological examination that detects CMV inclusion bodies. However, due to its high sensitivity and ability to quantify the viral load, quantitative PCR testing is often preferred over histopathological examination [21, 22]. Furthermore, PCR is more convenient and faster to use for detecting CMV than histopathological examination. However, it is important to note that in some cases of CMV infection in immunocompromised patients, the viral load may be undetectable. Therefore, a negative PCR result does not exclude the possibility of CMV infection in these patients. In our case report, we did not use histopathological examination for CMV diagnosis due to it being widely unavailable in our country and the preferred method for CMV diagnosis in our centre is PCR.

The patient in this case presented with symptoms and signs of pneumonitis, including shortness of breath, cough, and decreased oxygen saturation of up to 88%. A thorax MSCT scan revealed ground-glass opacity and infiltrate in the right upper lobe lung area, as well as ground-glass opacity in the left upper lobe lung area. The patient had a history of kidney cancer and was undergoing anticancer therapy. Under these conditions, CMV PCR examination was performed to determine the aetiology of pneumonitis and the result was positive.

Management of CMV in immunocompetent patients is generally not necessary as the disease is often self-limited. However, in immunocompromised patients, antiviral therapy should be considered due to poor outcomes and increased risk of reactivation of infection when compared to immunocompetent patients. For example, a study conducted by Wang et al. that analysed 107 cancer patients with which CMV viremia were analysed showed that the mortality rate can be predicted from CMV viremia in cancer patients [9].

Several antivirals that have been approved and can be given to patients with CMV infection are cidofovir, ganciclovir, valganciclovir, or foscarnet [23]. Currently, first-line therapy for CMV infection is either intravenous ganciclovir or oral valganciclovir. Therapy should be initiated at full doses and adjusted according to renal function. It should be continued until symptoms resolve, and the CMV deoxyribonucleic acid (DNA) load becomes undetectable. If CMV is detected after 2 weeks of therapy, maintenance therapy may be considered [9, 23]. The patient, in this case, received intravenous ganciclovir at a dose of 10 mg/kg/24 hours for 14 days. After 14 days of treatment with intravenous ganciclovir, the patient's symptoms resolved. The patient was then discharged and received oral valganciclovir as maintenance treatment for 1 week.

An important question is whether CMV prophylaxis should be administered in cancer patients prior to cancer treatments. Serological tests are generally used to determine serostatus before organ transplantation for risk stratification of CMV infection. In our opinions, if the serological tests show evidence of active CMV infection or reactivation (elevated levels of specific CMV antibodies), CMV prophylaxis can be considered. However, currently, there are limited data on CMV infection in cancer patients regarding serology testing and prophylaxis [24]. Guideline from the Infectious Diseases Working Party of German Society for Hematology and Medical Oncology (DGHO) showed no evidence for CMV prophylaxis but CMV reactivation monitoring should be conducted in patients receiving alemtuzumab [24].

## 4. Conclusion

This case highlights the increased severity of CMV infection in clear cell renal cell carcinoma compared to that in immunocompetent individuals. Cancer patients who are undergoing treatment are at a higher risk for developing opportunistic infections such as from CMV, which can result in morbidity and mortality. Therefore, healthcare professionals should be aware of the possibility of CMV infection or reactivation in cancer patients and be prepared to diagnose and treat the infection, particularly in areas where the prevalence of CMV infection is high.

## Figures and Tables

**Table 1:** Laboratory examination results of the patient on the 4^th^ day of hospitalization.

Parameters (units)	Results	Reference range, adults
Hemoglobin (g/dL)	10.8	11.7–15.5
Hematocrit (%)	33	35–47
Leukocyte (/mm^3^)	7600	4500–11,000
Basophil (%)	0	0-1
Eosinophil (%)	1	1–3
Band neutrophil (%)	0	0–5
Segmented neutrophil (%)	65	50–70
Lymphocyte (%)	23	20–40
Monocyte (%)	11	4–8
Platelet (/mm^3^)	246000	150000–440000
Total protein (g/dL)	7.2	6.6–8.8
Albumin (g/dL)	4	3.5–5.2
Globulin (g/dL)	3.2	2.3–3.5
Total bilirubin (mg/dL)	0.54	0.1–1.2
Direct bilirubin (mg/dL)	0.17	≤0.2
Indirect bilirubin (mg/dL)	0.37	0.1–1.0
AST (U/L)	19	<35
ALT (U/L)	14	<41

## Data Availability

The data are not available for public viewing as it is part of a medical record.
